# Umbilical cord-derived mesenchymal stromal cells preserve endogenous insulin production in type 1 diabetes: a Phase I/II randomised double-blind placebo-controlled trial

**DOI:** 10.1007/s00125-023-05934-3

**Published:** 2023-05-24

**Authors:** Per-Ola Carlsson, Daniel Espes, Sofia Sisay, Lindsay C. Davies, C. I. Edvard Smith, Mathias G. Svahn

**Affiliations:** 1 Department of Medical Cell Biology, Uppsala University, Uppsala, Sweden; 2 Department of Medical Sciences, Uppsala University, Uppsala, Sweden; 3 Karolinska Trial Alliance, Karolinska University Hospital, Huddinge, Sweden; 4 Science for Life Laboratory, Department of Medical Cell Biology, Uppsala University, Uppsala, Sweden; 5 Science for Life Laboratory, Department of Medical Sciences, Uppsala University, Uppsala, Sweden; 6 NextCell Pharma AB, Huddinge, Sweden; 7 Department of Microbiology, Tumor and Cell Biology, Karolinska Institutet, Solna, Sweden; 8 Department of Laboratory Medicine, Biomolecular and Cellular Medicine, Karolinska Institutet, Huddinge, Sweden

**Keywords:** Advanced therapy medicinal product, Cell therapy, Clinical trial, Intervention, Mesenchymal stromal cells, Stem cells, Type 1 diabetes, Umbilical cord

## Abstract

**Aim/hypothesis:**

This study aimed to investigate the safety and efficacy of treatment with allogeneic Wharton’s jelly-derived mesenchymal stromal cells (MSCs) in recent-onset type 1 diabetes.

**Methods:**

A combined Phase I/II trial, composed of a dose escalation followed by a randomised double-blind placebo-controlled study in parallel design, was performed in which treatment with allogeneic MSCs produced as an advanced therapy medicinal product (ProTrans) was compared with placebo in adults with newly diagnosed type 1 diabetes. Inclusion criteria were a diagnosis of type 1 diabetes <2 years before enrolment, age 18–40 years and a fasting plasma C-peptide concentration >0.12 nmol/l. Randomisation was performed with a web-based randomisation system, with a randomisation code created prior to the start of the study. The randomisation was made in blocks, with participants randomised to ProTrans or placebo treatment. Randomisation envelopes were kept at the clinic in a locked room, with study staff opening the envelopes at the baseline visits. All participants and study personnel were blinded to group assignment. The study was conducted at Karolinska University Hospital, Stockholm, Sweden.

**Results:**

Three participants were included in each dose cohort during the first part of the study. Fifteen participants were randomised in the second part of the study, with ten participants assigned to ProTrans treatment and five to placebo. All participants were analysed for the primary and secondary outcomes. No serious adverse events related to treatment were observed and, overall, few adverse events (mainly mild upper respiratory tract infections) were reported in the active treatment and placebo arms. The primary efficacy endpoint was defined as Δ-change in C-peptide AUC for a mixed meal tolerance test at 1 year following ProTrans/placebo infusion compared with baseline performance prior to treatment. C-peptide levels in placebo-treated individuals declined by 47%, whereas those in ProTrans-treated individuals declined by only 10% (*p*<0.05). Similarly, insulin requirements increased in placebo-treated individuals by a median of 10 U/day, whereas insulin needs of ProTrans-treated individuals did not change over the follow-up period of 12 months (*p*<0.05).

**Conclusions/interpretation:**

This study suggests that allogeneic Wharton’s jelly-derived MSCs (ProTrans) is a safe treatment for recent-onset type 1 diabetes, with the potential to preserve beta cell function.

**Trial registration:**

ClinicalTrials.gov NCT03406585

**Funding:**

The sponsor of the clinical trial is NextCell Pharma AB, Stockholm, Sweden.

**Graphical Abstract:**

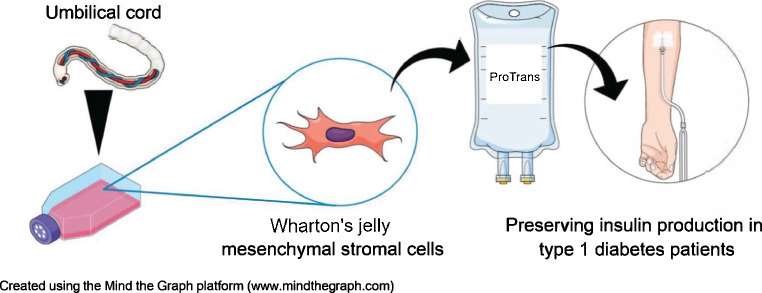

**Supplementary Information:**

The online version of this article (10.1007/s00125-023-05934-3) contains peer-reviewed but unedited supplementary material.


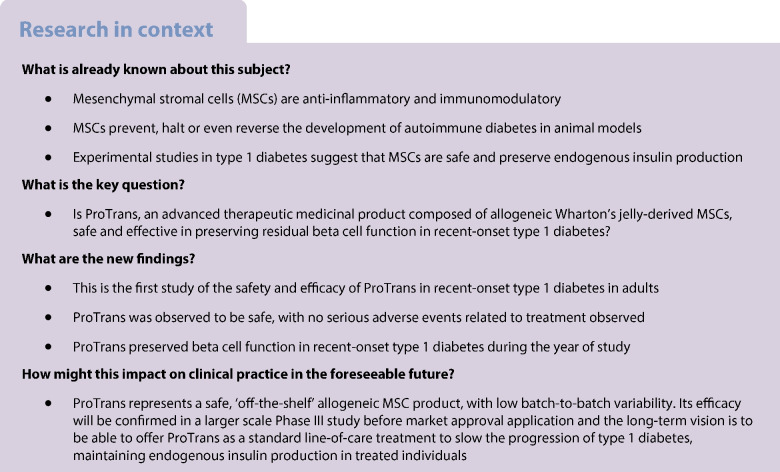


## Introduction

Type 1 diabetes is an autoimmune disorder, affecting 8.4 million people worldwide in 2021 [1]. Exogenous insulin, used for the treatment of type 1 diabetes since 1922, remains the current standard of care. At diagnosis, beta cell function is regularly seen to have decreased to 10–30% of normal levels, representing a challenge for therapeutic intervention to reverse disease status. Therefore, a focus has been placed on strategies for retaining and controlling endogenous insulin, thereby substantially decreasing the risk of acute and long-term complications [2–4].

Despite great advances in insulin formulations and medical technology devices, people living with type 1 diabetes still have an increased risk of complications and, dependent on age at diagnosis and sex, have a shortened life span by 10–18 years [5]. The recent approval of teplizumab for delaying onset of stage 3 type 1 diabetes has provided hope for the development of novel interventive strategies for disease prevention [6]. Despite this, translational research for the development of new interventive therapies that may reverse disease status or slow its progression remains sparse. Drug candidates are still primarily in the pilot stage, with research still focused on insulin or glucagon.

Mesenchymal stromal cells (MSCs) represent a novel approach to diabetes treatment. These multipotent progenitor cells possess innate immunomodulatory, proangiogenic and antifibrotic properties [7]. They can be found in many connective tissues but are most frequently obtained for clinical usage from the bone marrow, adipose tissue or umbilical cord. MSCs obtained from the gelatinous substance within the umbilical cord, Wharton’s jelly MSCs (WJMSCs), are highly attractive as a cell source because of their rich stemness, well-defined characteristics, abundance within the tissue and rapid proliferation. The ease of accessibility of this tissue and the immunomodulatory properties of its resident MSCs lend themselves for use in allogeneic ‘off-the-shelf’ therapies [8–12].

MSCs have been exploited for their immunomodulatory capacity in numerous diseases including autoimmune disorders such as multiple sclerosis [13] and systemic lupus erythematosus (SLE [14]). While many in vivo murine studies have been conducted to evaluate the potential therapeutic benefit of MSCs in type 1 diabetes (reviewed in [15]), few clinical trials have been reported, with only two of these studies evaluating the use of allogeneic MSCs, and no studies evaluating a pooled allogeneic MSC product [16–19].

In this study we generated a drug product from culture-expanded and pooled WJMSCs, ProTrans, under the classification of an advanced therapy medicinal product (ATMP). The aim of this study was to evaluate the safety and efficacy of i.v. infusion of this ‘off-the-shelf’ allogeneic WJMSC product in individuals recently diagnosed with type 1 diabetes.

## Methods

### Approval

This study (clinicaltrialsregister.eu registration no. 2017-002766-50) was approved by the Swedish Ethical Review Authority (Dnr 2017/1533-31/2) and the Swedish Medical Products Agency (Dnr 5.1-2017-56212) and registered at ClinicalTrials.gov (NCT03406585). The study was conducted in accordance with the Declaration of Helsinki.

### Trial design

This was a Phase I/II single-site study, sponsored by NextCell Pharma, Stockholm, Sweden, and conducted at Karolinska University Hospital, Stockholm, Sweden. The study protocol was published in advance [20]. The first part (part A) was a dose-escalating study of nine male participants (three different doses of allogeneic WJMSC [ProTrans; NextCell Pharma, Sweden] delivered as an i.v. infusion; 25, 100 and 200 million cells; *n*=3 participants for each dose), 18–40 years of age, with newly diagnosed type 1 diabetes (diagnosed <2 years before enrolment). The second part (part B) was a randomised double-blind placebo-controlled study comparing ProTrans treatment (200 million cells [dose selected based on results of part A]; *n*=10 participants) with placebo (vehicle control; *n*=5 participants) in individuals 18–40 years of age, both male and female, newly diagnosed with type 1 diabetes. Both the participants and the study personnel were blinded to the treatment. Visits and investigations were scheduled according to details presented in electronic supplementary material (ESM) Table 1. All recruitment and follow-up visits were performed between quarter 1 in 2018 and quarter 2 in 2020. Karolinska Trial Alliance, Stockholm, Sweden were contracted to monitor the study.

Randomisation was performed with a web-based randomisation system with a randomisation code created prior to the start of the study. The randomisation was made in blocks, with participants randomised to batch 1 ProTrans, batch 2 ProTrans or placebo treatment. Randomisation envelopes were kept at the clinic in a locked room, with study staff opening a randomisation envelope at the baseline visit.

The primary safety outcome was evaluated through the registration of adverse events (AEs). The primary efficacy variable was the comparison of the intervention (ProTrans) vs placebo as measured by the ∆-change in C-peptide AUC (0–120 min) for a mixed meal tolerance test (MMTT) at day 372 following ProTrans/placebo infusion when compared with pretreatment. Secondary outcome measures assessed the number of insulin-independent participants and the number of participants with daily insulin needs <0.25 U/kg, insulin requirement/kg body weight, HbA_1c_ at days 187 and 262 after treatment, glucose variability (mean amplitude of glycaemic excursions and glycaemic lability index) and hypo/hyperglycaemia duration as assessed using a continuous glucose monitoring (CGM) system, ∆-change of levels of C-peptide compared with baseline and number of participants with peak C-peptide >0.2 nmol/l in response to MMTT, at day 372.

### Enrolment and randomisation

Participants were recruited from Uppsala University Hospital, Uppsala, Sweden, collaborating hospitals and by recruitment advertising. The participants were informed about the study by the principal investigator (P-OC) or the co-investigators at Karolinska Trial Alliance’s Phase I clinic, Karolinska University Hospital. All participants were supplied with oral and written information on the study and provided written, informed consent.

Inclusion criteria were a diagnosis of type 1 diabetes <2 years before enrolment, age 18–40 years and a fasting plasma C-peptide concentration >0.12 nmol/l. Exclusion criteria were BMI >30 kg/m^2^, weight <50 kg or >100 kg, unstable cardiovascular status, active and chronic infections such as tuberculosis, HIV, hepatitis B or C or treponema pallidum infection, ongoing systemic immunosuppressive therapy, demyelinating disease, pregnancy or lactation (women), malignancy, glucose-lowering therapies other than insulin, a diagnosis of kidney disease defined as an eGFR of less than 80 ml/min per 1.73 m^2^ body surface, proliferative retinopathy, or known hypersensitivity reaction to excipients (i.e. DMSO).

In part A of the study, only male participants were included since studies of possible HLA immunisation formed part of the safety analysis. Three participants were treated with low-dose ProTrans (25 million cells), followed by three participants receiving 100 million cells and three participants receiving 200 million cells. Based on the safety results in part A, women were allowed to participate in part B. Randomisation in part B of the study was performed using a web-based randomisation system, in blocks without stratification to either batch 1 ProTrans, batch 2 ProTrans or placebo treatment. All female participants were required to agree to use acceptable birth control (defined as methods with a failure rate of <1% per year when used correctly) to participate.

### Investigational product and treatment

The investigational product ProTrans is a clinical-grade cell suspension of MSCs procured from donated Wharton’s jelly tissue and expanded in adherent culture over approximately 4–5 weeks (a maximum of three passages) according to Good Manufacturing Practice (GMP). Umbilical cord tissue donors were provided with both written and oral information regarding tissue donation by the healthcare provider. Informed written consent was provided prior to tissue collection. Expanded cells from five donors were pooled at the point of product formulation prior to cryopreservation. Release characterisation criteria state that the investigational product consists of MSCs expressing (>70%) CD73, CD90, CD105 cell surface antigens and negative (<5%) for CD14, CD19, CD34, CD45 and HLA-DR. This is in line with the International Society for Cell and Gene Therapy’s minimal criteria for MSC identity [21]. The product is quality controlled to ensure absence of microbial contamination, endotoxin and mycoplasma, viability (>80%) and an ability to attach to plastic. The cell product is cryopreserved at a concentration of 100 million cells in a 5 ml solution of 5% (wt/vol.) human serum albumin supplemented with 10% (vol./vol.) DMSO.

The placebo consisted of excipients as above without cell product (5% wt/vol. human serum albumin supplemented with 10% vol./vol. DMSO) and was produced in accordance with procedures and materials used by the manufacturer in the preparation of the investigational product.

ProTrans/placebo cryobags cells were thawed bedside in a 37°C water bath. The thawed ProTrans/placebo (5 ml solution) was transferred to a 100 ml saline (154 mmol/l NaCl) infusion bag using a transfer spike. The diluted product was infused at a rate of 5.25 ml/min, equating to 5 million cells/min. Where a dose of 200 million cells or placebo was used, the participant was infused with 210 ml of diluted solution at the same rate as described above (two consecutive infusions of 105 ml). The 25 million cell dose was prepared as described above but the diluted product was infused at a rate of 1.31 ml/min; the infusion was stopped after 20 min resulting in the participant receiving 26.25 ml cell suspension.

### Clinical study procedures

All visits were performed in the morning (08:00–10:00 hours) after participants had fasted overnight. Physical examinations were conducted, as well as electrocardiography, BP and pulse measurements. An ophthalmological examination with retinal inspection was performed by a specialised ophthalmologist at visits 1, 4 and 8 (see ESM Table 1). MMTTs (Nestlé Resource protein, Nestlé Health Science, Vevey, Switzerland; 6 ml/kg, maximal dose 360 ml) were used to assess residual beta cell function, and venous blood samples were taken for glucose and C-peptide analyses at 0, 15, 30, 60, 90 and 120 min. Exogenous insulin requirements were assessed at study visits based on the participants’ recorded doses during the three consecutive days prior to visit. Mean daily insulin requirements were calculated based on these recordings. A participant-blinded system for CGM was performed for 72 h with the Dexcom G4 system (Dexcom, USA) to assess metabolic control and glucose variability. Metabolic control was optimised for the participants at every visit during the study with the aim of an optimal target glucose range of 4.4–7.2 mmol/l (80–130 mg/dl).

Routine laboratory variables, including serum GAD65 and IA-2 antibodies, and chronic infection variables were analysed at the Central Clinical Chemistry Laboratory, Karolinska University Laboratory, Stockholm, Sweden. Titres of HLA antibodies were determined by LABScreen Mixed Class I and II (Thermo Fisher Scientific, USA) at the Clinical Immunology Laboratory, Karolinska University Laboratory. HLA class II genotype determination was conducted by Gendia (Belgium).

### Data safety monitoring board

A data safety monitoring board (DSMB) consisting of three physicians with medical and scientific expertise in diabetology and cellular therapy was appointed by the sponsor to advise the principal investigator during the study and to recommend whether to continue, modify, or stop the investigation.

### Statistical analysis and sample size

In total, 24 participants were included, with nine participants in the dose escalation arm to assess safety (part A). The randomised part B (2:1) included ten participants treated with one of two batches of ProTrans and five participants who received the placebo control. For calculation of participant numbers, part B was considered to contain three randomised groups (placebo, batch 1 ProTrans and batch 2 ProTrans), and not to include any data from part A. *N*=5 per group provided a power of 95% and ANOVA of the three groups, allowing post hoc comparison between groups. The safety data, with reported AEs, were evaluated and compared between groups with respect to the number of participants affected (Fisher’s exact test) and total frequency in the different groups (negative binomial regression). Efficacy endpoints were evaluated using Student’s unpaired two-tailed *t* test for parametric data and Mann–Whitney test for non-parametric data. Normality was evaluated using the Shapiro–Wilk test and equal variances using the *F* test. Treatment difference was estimated with a 95% CI.

## Results

### Participant characteristics

In part A of the study, all participants were male, an average of 13 months after type 1 diabetes diagnosis at time of inclusion and had a BMI of 20–25 kg/m^2^ (Table 1). In part B, eight men and seven women were included, with an average of 12 months since type 1 diabetes diagnosis and with a BMI of 20–25 kg/m^2^ (Table 1). None of the participants included in either study part were smokers. One participant in part B decided to leave the study after 6 months of follow-up due to low motivation. All other included participants completed the study (ESM Fig. 1). No baseline characteristics differed between the groups in part A (Table 2).

**Table 1:** Characteristics of participants

Characteristic	Part A			Part B	
	Low dose (*N*=3)	Medium dose (*N*=3)	High dose (*N*=3)	Placebo (*N*=5)	ProTrans (*N*=10)
Age (years)	24±3	24±1	27±1	31±9	31±4
Sex (no. female/no. male)	0/3	0/3	0/3	3/2	4/6
Time since diagnosis (years)	1.1±0.0	1.1±0.1	0.8±0.7	1.0±0.3	1.0±0.7
Weight (kg)	75.1±10.4	80.2±8.1	75.7±2.7	67.5±12.6	73.9±9.3
BMI (kg/m^2^)	22.5±2.5	24.2±2.5	23.7±1.2	23.5±2.2	24.2±2.9

Data are presented as median ± SD

**Table 2:** Characteristics pre treatment and 1 year post treatment with ProTrans for participants included in part A of the study

Characteristic	Pre treatment			1 year post treatment		
	Low dose (*N*=3)	Medium dose (*N*=3)	High dose (*N*=3)	Low dose (*N*=3)	Medium dose (*N*=3)	High dose (*N*=3)
GAD65 (*n*/*N*)	2/3	2/3^a^	2/3	2/3	2/3	2/3
IA-2 (*n*/*N*)	2/3	1/3^a^	1/3	2/3	1/3	1/3
GAD65 and IA-2 (*n*/*N*)	1/3	1/3^a^	1/3	1/3	1/3	1/3
GAD65 or IA-2 (*n*/*N*)	3/3	2/3^a^	2/3	3/3	2/3	2/3
HbA_1c_ (mmol/mol)	42±5	44±12	47±14	43±2	41±12	40±4
HbA_1c_ (%)	6.0±0.7	6.2±1.7	6.5±1.9	6.1±0.3	5.9±1.7	5.8±0.6
Glucose (mmol/l)	6.1±0.9	6.4±1.3	6.7±0.2	6.3±1.1	6.6±1.9	6.8±0.4
Time above optimal glucose range (%)^b^	21±15	29±23	31±7	32±15	34±30	35±11
Time within optimal glucose range (%)^b^	68±7	52±13	67±8	43±11	44±16	57±8
Time below optimal glucose range (%)^b^	10±9	19±19	2±1	25±21	21±23	8±4
Insulin (U/day)	26.2±16.3	23.8±4.7	23.1±9.9	22.8±9.5	35.7±9.5	17.6±4
Insulin (U kg^−1^ day^−1^)	0.33±0.15	0.30±0.08	0.31±0.13	0.28±0.13	0.45±0.13	0.23±0.05
Fasting C-peptide (nmol/l)	0.31±0.01	0.27±0.01	0.26±0.1	0.28±0.09	0.19±0.02	0.31±0.07
MMTT AUC C-peptide (nmol/l) 2 h	1.07±0.34	0.60±0.07	0.67±0.13	0.79±0.32	0.55±0.14	0.63±0.06
MMTT C-peptide max (nmol/l) 2 h	1.6±0.7	0.9±0.1	1.0±0.2	1.0±0.4	0.7±0.2	0.9±0.1

Data are presented as median ± SD

^a^Data from one of the participants from visit 4

^b^Optimal range was defined as 4.4–7.2 mmol/l

Evaluation of baseline characteristics of the participants in part B of the study demonstrated that baseline C-peptide (MMTT AUC) was lower in the placebo group than in the active treatment group (Table 3; *p*=0.01). In line with this observation, fasting C-peptide was lower in the placebo group, although the difference was not significantly different from active treatment (Table 3; *p*=0.06).

**Table 3:** Characteristics at baseline for participants included in part B of the study

Characteristic	Pre treatment		1 year post treatment	
	Placebo (*N*=5)	ProTrans (*N*=10)	Placebo (*N*=5)	ProTrans (*N*=9)
GAD65 (*n*/*N*)	4/5	8/10	4/5	8/10
IA-2 (*n*/*N*)	4/5	8/10	4/5	8/10
GAD65 and IA-2 (*n*/*N*)	4/5	6/10	4/5	6/10
GAD65 or IA-2 (*n*/*N*)	4/5	10/10	4/5	10/10
HbA_1c_ (mmol/mol)	49±5	47±10	50±10	48±7
HbA_1c_ (%)	6.6±0.7	6.5±1.4	6.7±1.4	6.5±0.9
Glucose (mmol/l)^a^	6.3±1.4	6.3±1.5	6.8±1.8	7.7±0.8
Time above optimal glucose range (%)^b,c^	35±22	32±22	18±9	26±18
Time within optimal glucose range (%)^b,c^	39±9	49±17	71±20	71±19
Time below optimal glucose range (%)^b,c^	26±21	20±26	11±16	3±2
Insulin (U/day)	30±10	17±7	38±11	17±5
Insulin (U kg^−1^ day^−1^)	0.46±0.20	0.23±0.09	0.55±0.18	0.23±0.06
Fasting C-peptide (nmol/l)	0.16±0.05	0.32±0.19	0.14±0.13	0.33±0.19
MMTT AUC C-peptide (nmol/l) 2 h	0.40±0.22^d^	0.77±0.28	0.25±0.22	0.71±0.25
MMTT C-peptide max (nmol/l) 2 h	0.62±0.44	1.00±0.37	0.33±0.26	0.93±0.28

Data are presented as median ± SD

^a^Missing data from three participants in the placebo group and one participant in the ProTrans group at 1 year

^b^Optimal range was defined as 4.4–7.2 mmol/l

^c^Missing data from one participant in the placebo group and one participant in the ProTrans group at 1 year

^d^Evaluation of baseline characteristics of the participants demonstrated that baseline C-peptide (MMTT AUC) was lower in the placebo group than in the active treatment group (*p*=0.01); no other significant differences between treatment groups were found for any of the variables evaluated

### ProTrans is safe with no serious adverse events or HLA immunisation related to treatment

All participants included in parts A (*n*=9) and B (*n*=10 receiving active treatment) of the study tolerated the infusion of MSCs well. Two participants reported AEs connected to infusion of the investigational product; one perceived the smell of corn for 72 h after infusion and one had a headache which self-resolved within 1 h of drug administration. No serious adverse events (SAEs) related to treatment were reported. In total, one SAE was reported, a female participant who became pregnant during the course of the trial; the study centre was notified after she terminated the pregnancy and the woman completed the trial. Of the total AEs, most were mild and transient and considered unlikely to be related to the investigational product (ESM Tables 2 and 3). There was no statistically significant difference between treatment and placebo group for any category of AE. Common cold, flu-like symptoms and upper respiratory tract infection AEs were all categorised as viral infections. No significant difference between placebo and active treatment was reported for viral infection-related AEs based on either the number of participants affected (*p*=0.33) or their total frequency (*p*=0.26). There was no observed HLA immunisation as measured by LABScreen Mixed Class I and II. Participants were also evaluated for change in weight before and post treatment at 12 months. Participants treated with ProTrans in part B saw a median (SD) weight loss of 0.09 (3.28) kg. In the placebo group a median (SD) weight gain of 4.28 (3.17) kg was seen.

### High doses of ProTrans maintain endogenous insulin production

#### Dose escalation

**Fig. 1:**
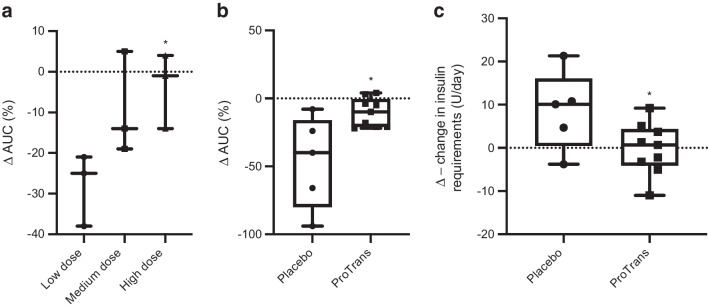
(**a**) Percentage Δ-change in C-peptide AUC (0–120 min) for the MMTT comparison between baseline (before treatment) and 12 months after treatment, at the day 372 visit. A comparison of participants treated in part A of the study (dose escalation study) was performed. Participants receiving high-dose ProTrans (*n*=3) demonstrated a maintenance of their % Δ AUC compared with participants treated with low-dose ProTrans (*n*=3; *p*=0.02, Mann–Whitney test). (**b**) % Δ-change in C-peptide AUC (0–120 min) for the MMTT comparison between baseline (before treatment) and 12 months after treatment, at the day 372 visit, for participants in part B of the study (ProTrans treatment, *n*=10; placebo, *n*=5). ProTrans showed a statistically significant effect compared with placebo (*p*=0.02, Mann–Whitney test). (**c**) Δ-change in daily insulin requirements in participants included in part B of the study, before treatment compared with 12 months after treatment. ProTrans showed a statistically significant effect compared with placebo (*p*=0.05, Student’s unpaired two-tailed *t* test). All data are presented as box and whisker plots min. to max. *p<0.05

Although part A of the study was principally designed for safety, a statistically significant dose-dependent efficacy of treatment was achieved, as assessed by ∆-change in C-peptide AUC. Treatment with ProTrans at a high dose (200 million cells) preserved beta cell function during the year of study when compared with low-dose treatment (25 million cells), as assessed by percentage ∆-change in C-peptide AUC (Fig. 1a; *p*<0.05; individual responses shown in ESM Fig. 2). These data were supported by correlative patterns for ∆-changes in fasting and peak C-peptide levels (Table 2).

#### Efficacy study

The randomised part B of the study was designed for the primary efficacy endpoint of Δ-change in C-peptide AUC at 12 months post treatment. While C-peptide levels were preserved in participants treated with ProTrans (200 million cells), declining by a median of only 10%, there was an median loss of 47% of C-peptide in the placebo-treated group (Fig. 1b; *p*<0.05; individual responses shown in ESM Fig. 3). As previously observed in part A of the study, these data demonstrating maintenance of endogenous insulin production with ProTrans treatment correlated with patterns of ∆-change in fasting and peak C-peptide levels across the treatment groups (Table 3). This correlated with the observation that insulin requirements increased in placebo-treated individuals by a median of 10 U/day, whereas insulin needs of ProTrans-treated individuals did not change over the follow-up period of 12 months (Fig. 1c; *p*<0.05).

Importantly, there was no statistical difference in efficacy between the two different batches of ProTrans product used in part B of the trial (*p*=0.96; data not shown).

All participants were receiving insulin treatment at inclusion in the trial, with none of them becoming insulin independent during the course of study (data not shown). However, when analysing the ∆-changes in insulin requirements during the year of study, insulin doses in participants treated with ProTrans did not change, while mean increases in insulin doses were observed in the placebo-treated group (Fig. 1c; *p*<0.05).

HbA_1c_ levels did not change during the course of the study in any of the groups (Tables 2, 3).

## Discussion

The prevalence of type 1 diabetes has surged in the last decade, partly due to non-genetic factors such as increased BMI and industrialisation [22], with adult-onset type 1 diabetes representing 70% of new cases worldwide [23–26]. Despite increasing demand on healthcare providers, and a growing understanding of the disease pathophysiology and risk factors, our fundamental approach to treatment remains the replacement of insulin. The use of cell therapies for the treatment of type 1 diabetes has focused on restoring physiological insulin production through regenerative or tissue engineering approaches to beta cell substitution [27]. Significant challenges remain, however, such as risk of teratoma formation with embryonic stem cells, low reprogramming efficiency and risk of rejection with induced pluripotent stem cells and necrosis of islet cells that have been transplanted into organs such as the liver [27].

The use of MSCs in the treatment of immune disorders, including autoimmune diseases, has demonstrated their potential as a safe interventive therapy for type 1 diabetes. The aim of this study was to assess the safety and efficacy of the WJMSC drug product, ProTrans, in maintaining endogenous insulin production to slow the progression of type 1 diabetes.

Previous clinical studies with MSCs have used various cell sources (e.g. bone marrow, fat or umbilical cord). MSCs from different sources have distinct properties and advantages based on production method, mode of administration, formulation and clinical indication for which they are employed [28].

Principally, type 1 diabetes clinical trials have been conducted using autologous MSCs. In addition to known limitations of autologous drug products, Davies et al [29] reported that comparative analysis of MSCs from individuals with type 1 diabetes and MSCs from healthy donors demonstrated distinct transcriptomic profiles with respect to immunomodulation, wound healing and haemocompatibility. The use of an allogeneic, healthy source of cells can overcome these potential safety and efficacy issues.

The umbilical cord has the advantage of generating large quantities of cells from an easily accessible tissue, allowing generation of a commercially viable batch size of product, without the need to age the cells in vitro to levels that may jeopardise their functionality or safety [30–32]. ProTrans also provides a distinct advantage as it is produced by pooling cells from five different donors. This reduces batch-to-batch variability and allows scalability of the product while maintaining safety and efficacy.

As ProTrans is an allogeneic cell therapy originating from multiple donors who are not HLA-matched; signs of HLA immunisation were investigated during dose escalation and only men were included. No HLA immunisation was seen on infusion of the product. With these safety data, women were included in part B of the study. Data from the efficacy study confirmed no HLA immunisation in the additional participants, indicating that the use of allogeneic cells did not influence safety with regard to histocompatibility antigen differences.

Of the doses tested, 25, 100 and 200 million cells, all met the safety requirements. AEs were reported in all dose groups but were mild to moderate. No significant differences in AEs were reported between the doses evaluated and no SAEs related to treatment were recorded. Despite the inclusion of only three participants in each dose group, the highest dose (200 million cells) significantly preserved endogenous insulin production during the first year after treatment. These data provided the preliminary evidence for choice of dose in part B of the study. ProTrans, unlike other cell products reported, uses a fixed dose of cells rather than a particular number of cells per kg body weight. This is the first time a significant effect on maintenance of endogenous insulin production has been documented using a fixed number of MSCs. Fixed dosing offers distinct advantages for commercialisation of a cell therapy product, as the batch of cells produced directly relates to the number of individuals who can be treated, with no wastage of cells or the need for post-release processing to count cells or formulate them according to an individual’s weight. This means that ProTrans can be a true, ‘off-the-shelf’ product, thawed at bedside with no waiting period for the individual being treated.

With the inclusion of ten participants within part B of the trial, it was necessary to use two batches of ProTrans product, derived from different umbilical cord donors. As part of the analysis in this study we compared participant response in terms of the primary endpoint using stratification based on the batch of product used. Both batches demonstrated no notable differences in safety or efficacy, suggesting low batch-to-batch variability of the product, a key feature for an allogeneic cell therapy.

The primary efficacy endpoint was ∆-change in C-peptide AUC at 12 months post treatment. As seen with the preliminary results of the dose escalation study, a single administration of ProTrans induced significant retention of endogenous insulin production compared with placebo. This was also reflected clinically, with the participants receiving ProTrans requiring no change in exogenous insulin treatment at 12 months compared with their respective baseline requirements. This was in contrast to placebo-treated participants, who exhibited the expected increase in insulin requirements with disease progression. It should be noted that a significantly higher level of C-peptide was evident pre treatment in the ProTrans-treated group than in the placebo control group (*p*=0.01; Table 3) and, for this reason, Δ-change in C-peptide AUC was used as the primary endpoint. No significant changes in weight of the participants were observed between the placebo and the ProTrans treatment groups. Further studies with larger cohorts are under development to confirm the impact of ProTrans on slowing disease advancement. Preserved endogenous insulin production has, in some studies, been shown to improve metabolic control, lowering HbA_1c_ and the risk of hypoglycaemic events [3]. Within the context of cell therapy trials, the use of HbA_1c_ as a clinical indicator of response and beta cell function is treated with caution. This is principally because exogenous insulin and standard line of care are continued throughout the course of the trials, maintaining HbA_1c_ <53 mmol/mol (7%) [33]. As such, it was not surprising that in this study no differences between study groups regarding HbA_1c_ or percentage optimal glucose values, and no severe (assisted) hypoglycaemic events, were reported. A limitation of this study with regard to the evaluation of average glucose levels between the placebo- and the ProTrans-treated groups at 12 months’ follow-up is that data were missing for this variable for three of the five participants in the placebo arm.

The lack of difference in HbA_1c_ between the groups also suggests that treatment effects were not merely a result of alleviated metabolic stress on beta cells, a phenomenon previously reported to slow down the progression to total insulin deficiency [34]. Experimentally, many different mechanisms have been reported for the role of MSCs in preserving beta cell function, including systemic immunomodulatory effects through TNF-α-stimulated gene/protein 6 [34] and IL-6 [35]. The mode of MSC administration is an important consideration in evaluating outcomes of type 1 diabetes clinical trials. In most studies reported to date, both preclinical and clinical, an i.v. route of delivery has been used [18], although there are also examples of intra-arterial delivery or local injections into the pancreas [36]. In this study we used the i.v. route, implying that the treatment effect would be mediated by systemic immune responses, rather than locally within the pancreas. MSCs infused intravenously trigger a domino effect of responses employing both the innate and the adaptive arms of the immune system, starting with the binding of complement through to the induction of regulatory T cells, to maintain the therapeutic effect we see here at 12 months post treatment [37–41]. These effects are seen in the context of multiple clinical indications that are maintained far beyond the presence of the infused MSCs, which are cleared within days [42, 43].

The data presented here support growing evidence that MSCs represent an attractive interventive therapy for slowing the progression of type 1 diabetes. An i.v. route was also employed in a previous study in which autologous bone marrow-derived MSCs were administered to adults newly diagnosed with type 1 diabetes [18]. This randomised study also showed that the treatment was safe and preserved endogenous insulin production at 1 year of follow-up, with unchanged HbA_1c_ levels. Other studies have also provided preliminary evidence that MSCs may be able to improve diabetic status in certain cohorts. Two studies using WJMSCs that were infused intravenously in 15 and 27 participants, respectively, reported remarkable results, with a reversal of insulin requirements in several treated participants post-infusion [16, 17]. Furthermore, in the Hu et al study [16], reduced HbA_1c_ levels and increased endogenous insulin production were seen at 24 months’ post treatment. Although they should be treated with caution and require reproduction, these data hint at the growing potential of MSC therapy as we move towards standardising the clinical trial design, participant cohort characteristics and the production methods employed in cell therapy [28]. Other studies have combined MSCs with other treatments (i.e. combined adipose-derived MSCs with vitamin D supplementation [44]) or WJMSCs with autologous bone marrow transplantation [45], making interpretation of the MSC effect more difficult.

The long-term therapeutic effect of a single infusion of MSCs remains to be ascertained. Participants from this study will be monitored for an additional 4 years to investigate the safety and long-term efficacy of ProTrans treatment. Since the natural course of type 1 diabetes is to lose most endogenous insulin production during the first 5 years [46], it will be of utmost interest to investigate whether the treatment effect is preserved and to determine the impact on metabolic control when differences in endogenous insulin production are magnified.

We conclude that ProTrans, composed of WJMSCs, is safe in the tested dose range of 25–200 million cells when administered intravenously, and that a single treatment in adults recently diagnosed with type 1 diabetes with 200 million cells results in preservation of their endogenous insulin for at least a year. Long-term follow-up is ongoing to investigate treatment safety and efficacy persists and to determine whether repeated treatment with ProTrans will be necessary for maintaining endogenous insulin production, thereby slowing disease progression and reducing the risk of type 1 diabetes-associated complications.

## Supplementary Information

Below is the link to the electronic supplementary material.

Supplementary file1 (PDF 473 KB)

## Data Availability

The datasets generated and/or analysed during the study are available from the corresponding author on reasonable request.

## Abbreviations

AE: Adverse event
ATMP: Advanced therapy medicinal product
CGM: Continuous glucose monitoring
MMTT: Mixed meal tolerance test
MSC: Mesenchymal stromal cell
SAE: Serious adverse event
SLE: Systemic lupus erythematosus
WJMSC: Wharton’s jelly mesenchymal stromal cell
