# Correction: Umbilical cord-derived mesenchymal stromal cells preserve endogenous insulin production in type 1 diabetes: a Phase I/II randomised double-blind placebo-controlled trial

**DOI:** 10.1007/s00125-025-06371-0

**Published:** 2025-02-25

**Authors:** Per‑Ola Carlsson, Daniel Espes, Sofia Sisay, Lindsay C. Davies, C. I. Edvard Smith, Mathias G. Svahn

**Affiliations:** 1 Department of Medical Cell Biology, Uppsala University, Uppsala, Sweden; 2 Department of Medical Sciences, Uppsala University, Uppsala, Sweden; 3 Karolinska Trial Alliance, Karolinska University Hospital, Huddinge, Sweden; 4 Science for Life Laboratory, Department of Medical Cell Biology, Uppsala University, Uppsala, Sweden; 5 Science for Life Laboratory, Department of Medical Sciences, Uppsala University, Uppsala, Sweden; 6 NextCell Pharma AB, Huddinge, Sweden; 7 Department of Microbiology, Tumor and Cell Biology, Karolinska Institutet, Solna, Sweden; 8 Department of Laboratory Medicine, Biomolecular and Cellular Medicine, Karolinska Institutet, Huddinge, Sweden

**Correction: Diabetologia (2023) 66:1431-1441**

10.1007/s00125-023-05934-3

Unfortunately, two entries were incorrect in Table 2. The proportion of participants in the high-dose group with IA-2 antibodies 1 year post treatment was incorrectly reported as 2/3 instead of 1/3. Similarly, the proportion of participants in the same group with GAD65 or IA-2 autoantibodies was incorrectly reported as 3/3 instead of 2/3. The authors are confident that these errors do not compromise the study design, analysis, interpretation or conclusions. The corrected table is shown here. The original article has been corrected.

**Table 2:** Characteristics pre treatment and 1 year post treatment with ProTrans for participants included in part A of the study

Characteristic	Pre treatment			1 year post treatment		
	Low dose (*N*=3)	Medium dose (*N*=3)	High dose (*N*=3)	Low dose (*N*=3)	Medium dose (*N*=3)	High dose (*N*=3)
GAD65 (*n*/*N*)	2/3	2/3^a^	2/3	2/3	2/3	2/3
IA-2 (*n*/*N*)	2/3	1/3^a^	1/3	2/3	1/3	1/3
GAD65 and IA-2 (*n*/*N*)	1/3	1/3^a^	1/3	1/3	1/3	1/3
GAD65 or IA-2 (*n*/*N*)	3/3	2/3^a^	2/3	3/3	2/3	2/3
HbA_1c_ (mmol/mol)	42 ± 5	44 ± 12	47 ± 14	43 ± 2	41 ± 12	40 ± 4
HbA_1c_ (%)	6.0 ± 0.7	6.2 ± 1.7	6.5 ± 1.9	6.1 ± 0.3	5.9 ± 1.7	5.8 ± 0.6
Glucose (mmol/l)	6.1 ± 0.9	6.4 ± 1.3	6.7 ± 0.2	6.3 ± 1.1	6.6 ± 1.9	6.8 ± 0.4
Time above optimal glucose range (%)^b^	21 ± 15	29 ± 23	31 ± 7	32 ± 15	34 ± 30	35 ± 11
Time within optimal glucose range (%)^b^	68 ± 7	52 ± 13	67 ± 8	43 ± 11	44 ± 16	57 ± 8
Time below optimal glucose range (%)^b^	10 ± 9	19 ± 19	2 ± 1	25 ± 21	21 ± 23	8 ± 4
Insulin (U/day)	26.2 ± 16.3	23.8 ± 4.7	23.1 ± 9.9	22.8 ± 9.5	35.7 ± 9.5	17.6 ± 4
Insulin (U kg^−1^ day^−1^)	0.33 ± 0.15	0.30 ± 0.08	0.31 ± 0.13	0.28 ± 0.13	0.45 ± 0.13	0.23 ± 0.05
Fasting C-peptide (nmol/l)	0.31 ± 0.01	0.27 ± 0.01	0.26 ± 0.1	0.28 ± 0.09	0.19 ± 0.02	0.31 ± 0.07
MMTT AUC C-peptide (nmol/l) 2 h	1.07 ± 0.34	0.60 ± 0.07	0.67 ± 0.13	0.79 ± 0.32	0.55 ± 0.14	0.63 ± 0.06
MMTT C-peptide max (nmol/l) 2 h	1.6 ± 0.7	0.9 ± 0.1	1.0 ± 0.2	1.0 ± 0.4	0.7 ± 0.2	0.9 ± 0.1

Data are presented as median ± SD

^a^Data from one of the participants from visit 4

^b^Optimal range was defined as 4.4–7.2 mmol/l

