# Supplementary material for: Umbilical cord-derived mesenchymal stromal cells preserve endogenous insulin production in type 1 diabetes: a Phase I/II randomised double-blind placebo-controlled trial
Source: Diabetologia. 2023 May 24;66(8):1431–41. doi: 10.1007/s00125-023-05934-3 (PMC10317874; doi:10.1007/s00125-023-05934-3)
Supplement: Supplementary file 1 — Supplementary file1 (PDF 473 KB) [file 125_2023_5934_MOESM1_ESM.pdf]

ESM Table 1: Visit schedule

	**1**<br>Screening visit	**2**<br>Baseline visit	**3**<br>Treatment	**4**	**5**	**6**	**7**	**8**
Days	-14 -0	0	7<br>(+/- 3days)	37<br>(+/- 3days)	97<br>(+/- 7 days)	187<br>(+/- 14 days)	277<br>(+/- 14 days)	372<br>(+/- 14 days)
Informed consent	x							
Eligibility criteria	x							
Demography	x							
Medical history	x							
Concomitant medications	x	x	x	x	x	x	x	x
Baseline symptoms	x	x						
Adverse events		x	x	x	x	x	x	x
Optimising diabetes care	x	x	x	x	x	x	x	x
Randomisation		x						
Insulin requirement		x	x	x	x	x	x	x
MMTT		x						x
CGM		x						x
ProTrans/placebo infusion			x					
DTSQ		x						x
Immunology tests		x		x				x
Clinical chemistry	x	x		x	x	x	x	x
HLA class I genotype		x						
HbA1c	x	x		x	x	x	x	x

Pregnancy test (HCG)	x							
Vital signs (heart rate, BP)	x	x	x	x	x	x	x	x
Dispensing diary card		x	x	x	x	x	x	
Collecting diary card			x	x	x	x	x	x
Physical examination	x	x	x	x	x	x	x	x
Detailed neurological examination	x	x	x	x	x	x	x	x
Cardiovascular examination incl. ECG	x	x	x	x	x	x	x	x
Ophthalmological examination	x			x				x

ESM Table 2: Summary of all adverse events during both parts of the trial

MedDRA Preferred term	Grade I	Grade II	Grade III	Grade IV	Frequency
Headache	x				< 5 %
Nasopharyngitis	x				< 20 %
Nasopharyngitis		x			< 15 %
Nasopharyngitis			x		< 5 %
Influenza	x				< 5 %
Respiratory disorder	x				< 5 %
Food poisoning with vomiting	x				< 5 %
Gastroenteritis		x			< 5 %
Abdominal pain upper		x			< 5 %
Glomerular filtration rate decreased	x				< 10 %
Liver function test abnormal	x				< 5 %
Cystatin C	x				< 5 %
Electrocardiogram QTc interval prolonged	x				< 5 %
Tingling sensation	x				< 5 %
Anxiety	x				< 5 %
Nasal injury	x				< 5 %
Limb injury	x				< 5 %
Back pain	x				< 5 %
Conjunctivitis	x				< 5 %
Fatigue	x				< 5 %
Lymphadenopathy	x				< 5 %
Iron deficiency anemia	x				< 5 %
Cervix carcinoma stage 0	x				< 10 %
Pregnancy		x			< 5 %

ESM Table 3: Summary of all adverse events per group as reported

Part A	At infusion (within 24 h)	During 12 months	Patients affected
**Low dose (n=3)**			
Common cold	0	3	2
Tingling/numbness	0	1	1
Symptoms of stress	0	1	1
Elevated P-ASAT	0	1	1
Elevated P-LSAT	0	1	1
Food poison, vomit	0	1	1
**Medium dose (n=3)**			
Common cold	0	8	2
Unilateral conjunctival injection	0	1	1
Stomach flu	0	1	1
Lumbago	0	1	1
Erythema after freestyle libre	0	1	1
Pollen allergy	0	1	1
**High (high dose (n=3)**			
Common cold	0	4	2
Smell of corn	1	0	1
Headache	1	0	1
Chlamydia infection	0	1	1
GFR <80	0	1	1
Weight gain	0	1	1
Hair loss	0	1	1
Dry skin with eczema head	0	1	1
Numbness in hands while sleeping	0	1	1

Part B	At infusion (within 24 h)	During 12 months	Patients affected
**Placebo (n=5)**			
Common cold	0	2	2
Cervical cell dysplasia	0	1	1
GFR <80	0	1	1
Pain in stomach	0	1	1
**Active (n=10)**			
Common cold	0	9	7
Traumatic limb injury	0	1	1
Anaemia	0	1	1
Stomach flu	0	1	1
Decreased Cystatin C	0	1	1
Pregnancy (SAE)	0	1	1
Prolonged QTc time	0	1	1
Enlarged Lymph nodes	0	1	1
GFR <80	0	1	1
Cervical intraepithelial neoplasia	0	1	1
Tiredness	0	1	1

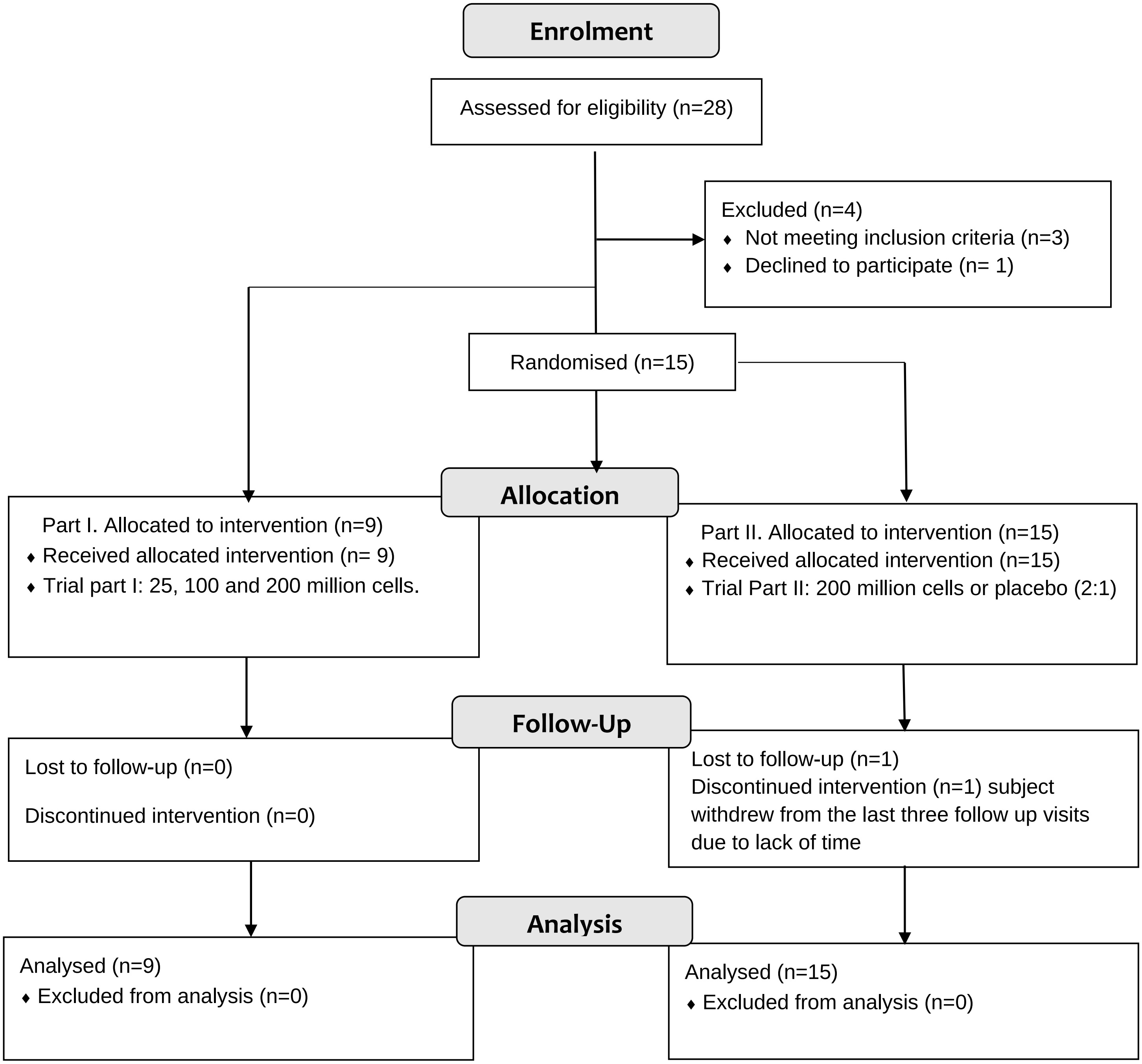

ESM Fig.1: Flow diagram of subjects in the clinical trial.

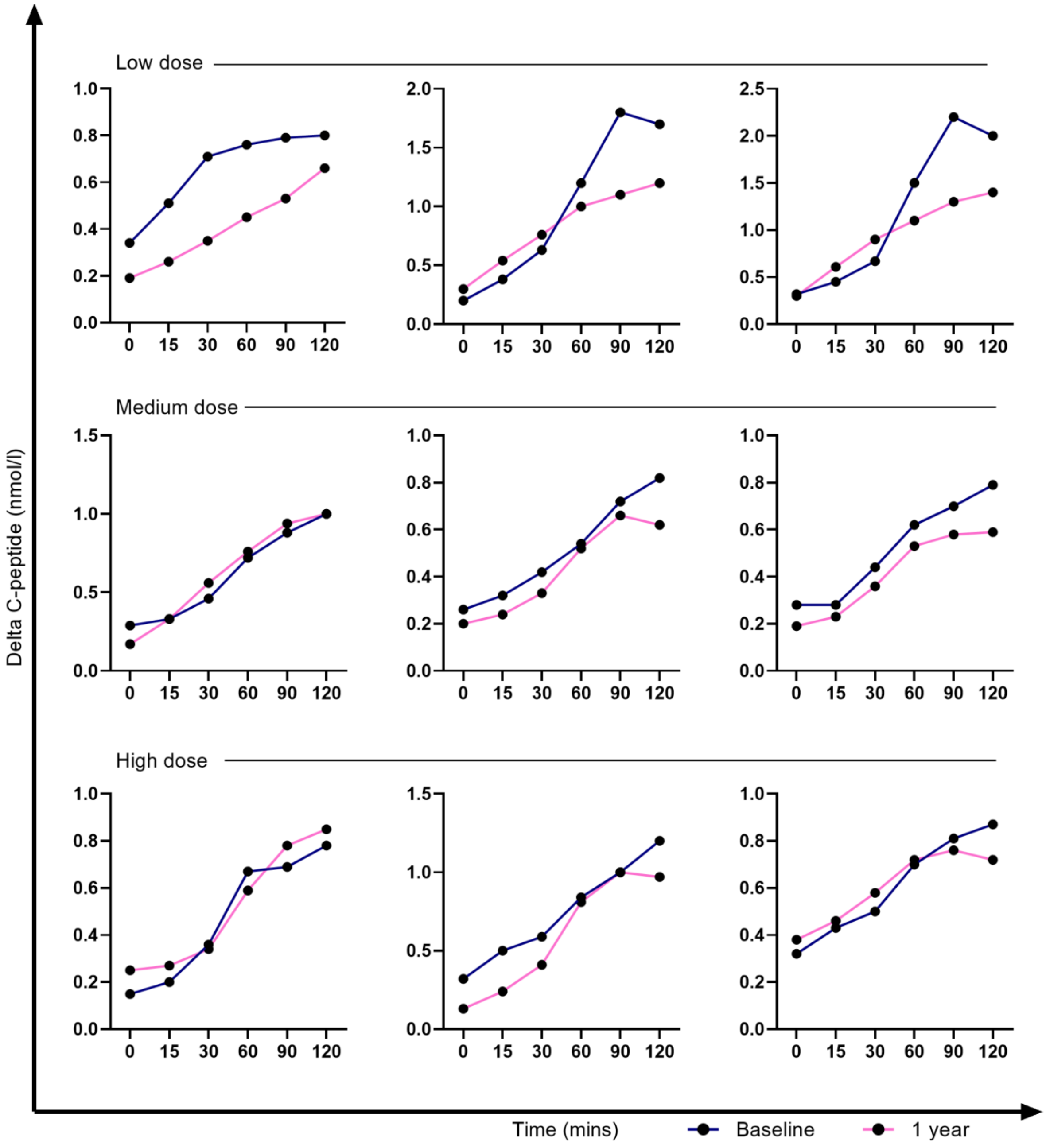

ESM Fig.2: C-peptide concentration during MMTT for each subject in part A. Comparison between baseline (before treatment) and 12 months after treatment

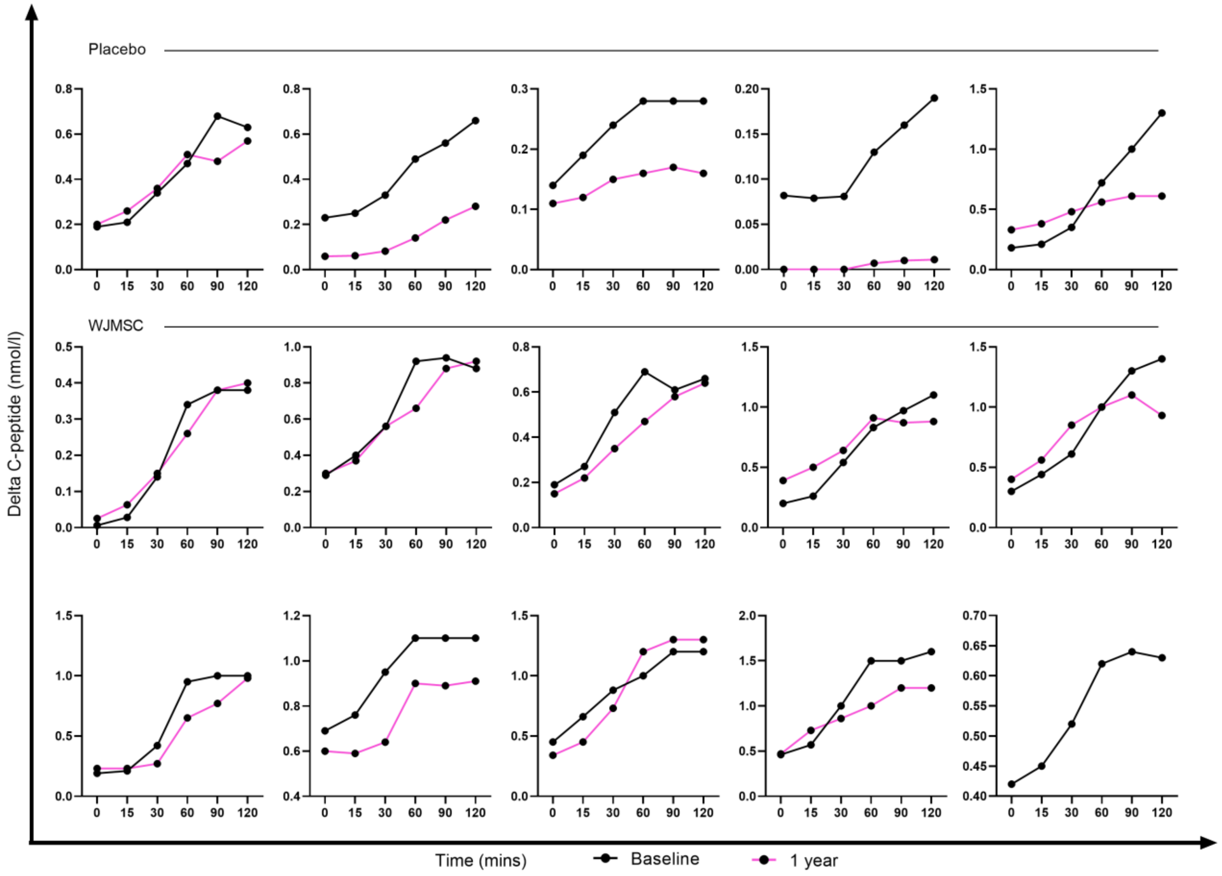

ESM Fig.3: C-peptide concentration during MMTT for each subject in part B. Comparison between baseline (before treatment) and 12 months after treatment
